# Enhanced Visualization of Choroidal Defects in Focal Choroidal Excavation With Near-Infrared Autofluorescence: Multimodal Imaging Study

**DOI:** 10.1167/tvst.15.9.10

**Published:** 2026-09-21

**Authors:** Yuta Nakanishi, Shin Kadomoto, Tomoaki Sakamoto, Takahiro Kogo, Yuki Muraoka, Masayuki Hata, Akitaka Tsujikawa

**Affiliations:** 1 Department of Ophthalmology and Visual Sciences, Kyoto University Graduate School of Medicine, Kyoto, Japan

**Keywords:** focal choroidal excavation, near-infrared fundus autofluorescence, short-wavelength fundus autofluorescence, dice similarity coefficient, fundus autofluorescence

## Abstract

**Purpose:**

To compare the spatial concordance of autofluorescence abnormalities on near-infrared fundus autofluorescence (NIRAF) and short-wavelength fundus autofluorescence (SWAF) with the area of focal choroidal excavation (FCE) defined on en face optical coherence tomography (OCT).

**Methods:**

In this retrospective cross-sectional observational study, a 6 × 6-mm en face swept-source OCT image centered on the fovea was used to define the structural FCE area on a choroidal slab. After image registration, areas of abnormal autofluorescence, including hypoautofluorescence and hyperautofluorescence, were delineated on SWAF and NIRAF. Spatial concordance between the en face OCT-defined FCE area and the abnormal autofluorescence areas was evaluated using the Dice similarity coefficient (DSC).

**Results:**

Twenty eyes of 20 patients with conforming-type FCE were included. The mean FCE area was 0.708 ± 0.571 mm^2^ on en face OCT, 1.174 ± 1.196 mm^2^ on NIRAF (*P* = 0.534 vs. en face OCT), and 0.152 ± 0.203 mm^2^ on SWAF (*P* < 0.001 vs. en face OCT). Spatial concordance with en face OCT was significantly greater for NIRAF (DSC, 0.654 ± 0.187) than for SWAF (DSC, 0.160 ± 0.214; *P* < 0.001). NIRAF detected abnormal autofluorescence corresponding to the FCE area in all eyes, whereas SWAF failed to detect any abnormality in six eyes (30%).

**Conclusions:**

FCE-associated abnormalities on NIRAF showed greater spatial concordance with the FCE area defined on en face OCT than those on SWAF.

**Translational Relevance:**

NIRAF offers a reproducible, noninvasive means of spatially mapping melanin-associated autofluorescence abnormalities, supporting more precise multimodal characterization of FCE.

## Introduction

Focal choroidal excavation (FCE) is a localized concavity of the choroid, most commonly in the macular region. It was first recognized by Jampol et al.^1^ in 2006, who described a unilateral, localized choroidal depression with near-normal overlying retinal architecture and preserved visual acuity on time-domain optical coherence tomography (OCT). The term “focal choroidal excavation” was later introduced by Margolis et al.^2^ in 2011 based on spectral-domain OCT findings. FCE is usually associated with good visual acuity and the lesion size often appears clinically stable; however, in a subset of eyes, it coexists with macular disorders such as central serous chorioretinopathy (CSC) or age-related macular degeneration (AMD), and the excavation may enlarge or be complicated by macular neovascularization (MNV) or CSC during the disease course.^3^^–^^6^ These observations suggest that FCE is not only an anatomic variant but also a potential site of choroidal vulnerability.

Previous studies have reported that the size of FCE is associated with the presence of concurrent macular pathology, and thus quantitative assessment of FCE morphology is considered important not only for descriptive characterization but also for evaluating disease activity and predicting prognosis.^7^ The diagnosis of FCE and its subtypes is made predominantly with OCT. Other imaging modalities—including fundus autofluorescence (FAF), fluorescein and indocyanine green angiography, and OCT angiography—are used only in a supplementary capacity, mainly to evaluate associated pathologies rather than to detect or characterize the excavation itself.^8^

FAF encompasses several imaging modalities that differ in excitation wavelength and predominant fluorophores. Among these, short-wavelength fundus autofluorescence (SWAF) is the most widely used in clinical practice and predominantly reflects lipofuscin-related autofluorescence from the retinal pigment epithelium (RPE). SWAF enables visualization of metabolic dysfunction and structural abnormalities such as RPE atrophy or excessive lipofuscin accumulation. Accordingly, SWAF has become an essential modality for delineating and quantifying atrophic lesions in geographic atrophy and is also widely used for the assessment of various retinal diseases, including retinitis pigmentosa.^9^ In FCE, SWAF may show varying degrees of hyper autofluorescence or hypo autofluorescence depending on alterations within the excavation or in the surrounding RPE. However, SWAF is not routinely used for the primary detection of the lesion of FCE.^8^

In recent years, near-infrared autofluorescence (NIRAF), a form of FAF that reflects melanin abnormalities in the RPE and choroid, has become available and is now used clinically for the detection of conditions associated with altered melanin, such as choroidal nevi and choroideremia.^10^^,^^11^ Because FCE involves localized deformation of the choroid, NIRAF may provide additional information on melanin-related autofluorescence changes associated with the excavation. Previous case-based and multimodal imaging reports have described clearer visualization of FCE lesions with NIRAF than with SWAF on visual assessment.^12^^,^^13^ These findings are biologically plausible because NIRAF is sensitive to melanin-associated signals in the RPE and choroid, whereas SWAF primarily reflects lipofuscin-related signals and may be attenuated by macular pigment in the foveal and parafoveal regions. Although previous case-based and multimodal imaging reports have suggested clearer visualization of FCE lesions with NIRAF than with SWAF, quantitative evidence regarding the spatial concordance between autofluorescence abnormalities and the structural extent of FCE remains limited, particularly when using registered en face OCT as an anatomical reference.

Building on these observations, we aimed to perform a registration-based quantitative comparison by aligning NIRAF and SWAF images with en face OCT and assessing the spatial concordance of autofluorescence abnormalities on each modality with the OCT-defined FCE area as the anatomical reference.

## Methods

### Study Design and Setting

This retrospective, observational study was conducted at Kyoto University Hospital. The study protocol was approved by the Institutional Review Board of Kyoto University Graduate School of Medicine (No. R0532). All procedures were performed in accordance with the Declaration of Helsinki. The requirement for informed consent was waived due to the retrospective nature of the study and the use of anonymized clinical data, as permitted by the Institutional Review Board.

### Patients

We included consecutive patients who first visited Kyoto University Hospital between July 2018 and March 2025 and were diagnosed with FCE. At the initial visit, all patients underwent best-corrected visual acuity measurement, color fundus photography, axial length measurement using the IOLMaster (Carl Zeiss Meditec, Dublin, CA, USA), spectral-domain OCT (Spectralis HRA+OCT; Heidelberg Engineering, Heidelberg, Germany), swept-source OCT en face imaging (PLEX Elite 9000; Carl Zeiss Meditec), and fundus autofluorescence imaging (SWAF and NIRAF; Spectralis HRA+OCT).

**Figure 1.**
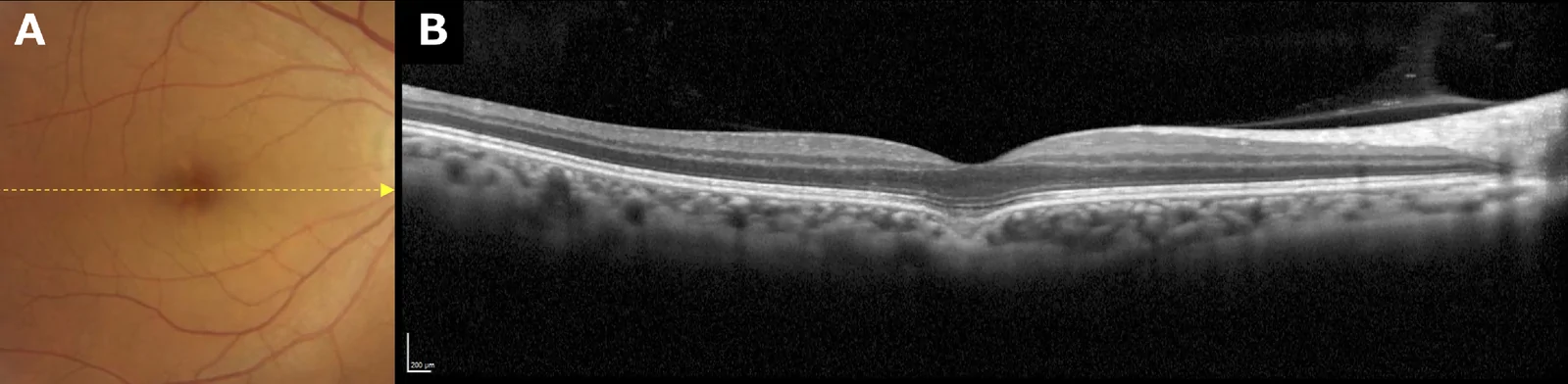
**Color fundus photograph and corresponding OCT B-scan of FCE.** (**A**) Color fundus photograph showing no remarkable fundus findings at the fovea. The *yellow line* indicates the OCT scan location. (**B**) Corresponding OCT B-scan along the *yellow line* in (**A**), demonstrating FCE.

FCE was defined and diagnosed by retina specialists as a circumscribed depression within the choroid on cross-sectional OCT, characterized by preservation of apposition between the photoreceptor outer segments and the RPE. Only conforming-type excavations were included (Fig. 1). Eyes with coexisting retinal fluid or excavations attributable to secondary causes such as inflammation, infection, or neovascular disease were excluded. We also excluded eyes with MNV, CSC, Vogt-Koyanagi-Harada disease, Best disease, or other conditions involving RPE or melanin abnormalities. In addition, eyes with poor-quality images were excluded.

### FCE Measurements Based on En Face OCT and FAF

A 6 × 6-mm en face OCT scan centered on the fovea was acquired using the PLEX Elite 9000. The FCE area was defined as the hyperreflective region on the en face OCT image at the choroidal slab between Bruch's membrane and the sclerochoroidal junction. The boundary was manually delineated on the en face image while referring to the corresponding B-scan OCT images to confirm lesion margins. Subfoveal choroidal thickness was defined as the distance from the outer border of Bruch's membrane to the sclerochoroidal interface.

**Figure 2.**
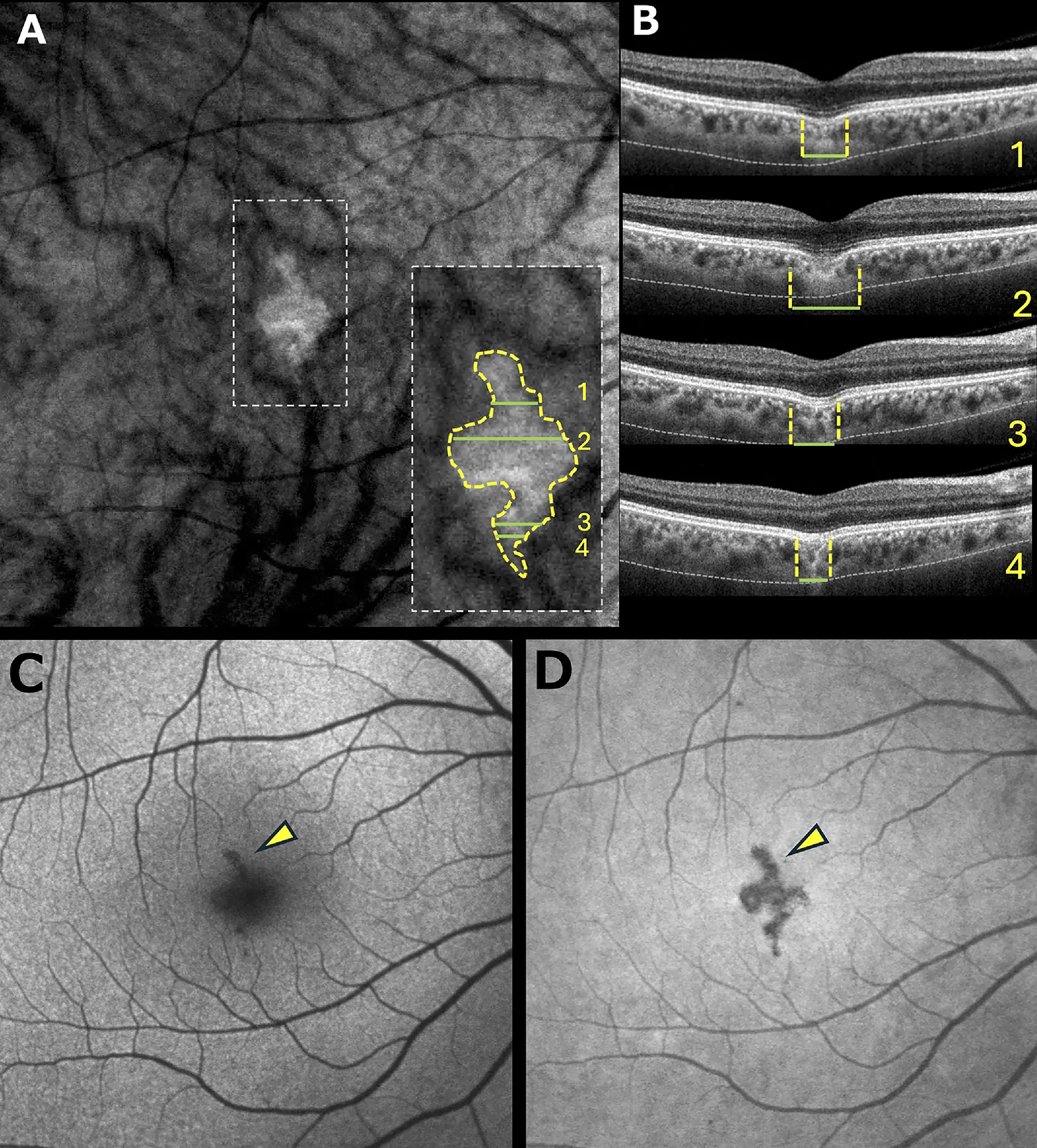
**Imaging-based definition of the FCE area for quantitative analysis.** (**A**) The FCE area was defined on the en face OCT image as a hyperreflective region (*yellow dashed outline*) and used as the reference for subsequent agreement analyses. The *green lines* indicate the positions of the B-scan sections shown in (**B**). (**B**) B-scan OCT sections (1–4) through the lesion demonstrate the location of the excavation. *Green lines* indicate the width of the excavation. SWAF (**C**) and NIRAF (**D**) images. *Yellow arrowheads* indicate abnormal autofluorescence regions, which were evaluated for spatial agreement with the reference FCE area defined on en face OCT.

SWAF and NIRAF were obtained with a confocal scanning laser ophthalmoscope using a 30° lens (field of view, 30° × 30°). (SWAF: 488-nm excitation; NIRAF: 820-nm excitation.) Areas of abnormal autofluorescence (hypoautofluorescence or hyperautofluorescence) on SWAF and NIRAF images were delineated for analysis (Fig. 2).

### Image Registration of En Face OCT and FAF Images

All measurements were performed after registering the FAF images to the en face OCT images. Image registration was performed to align the vasculature and anatomical landmarks on the SWAF and NIRAF images with those on the en face OCT image, thereby transforming the both autofluorescence images to correspond with en face OCT image. After initial scaling and rotation using an affine transformation, nonlinear image registration was performed in ImageJ version 1.52b (National Institutes of Health, Bethesda, MD, USA) using dedicated plugins. Using the Landmark Correspondences plugin, anatomically corresponding points (e.g., vessel bifurcations) were manually selected between the en face OCT image and each autofluorescence image (SWAF and NIRAF), and a nonlinear transformation was applied to each autofluorescence image to match these points, with the en face OCT image serving as the reference.^14^ A minimum of nine landmarks (e.g., retinal vessel bifurcations) were selected per image, and registration accuracy was visually confirmed by assessing the alignment of retinal vessels.

The FCE area on en face OCT and abnormal autofluorescence areas on registered SWAF and NIRAF images were delineated and measured in ImageJ. To correct for ocular magnification, axial length correction was applied to all measurements using the modified Bennett formula.^15^

### Inter-Modality Image Comparison

**Figure 3.**
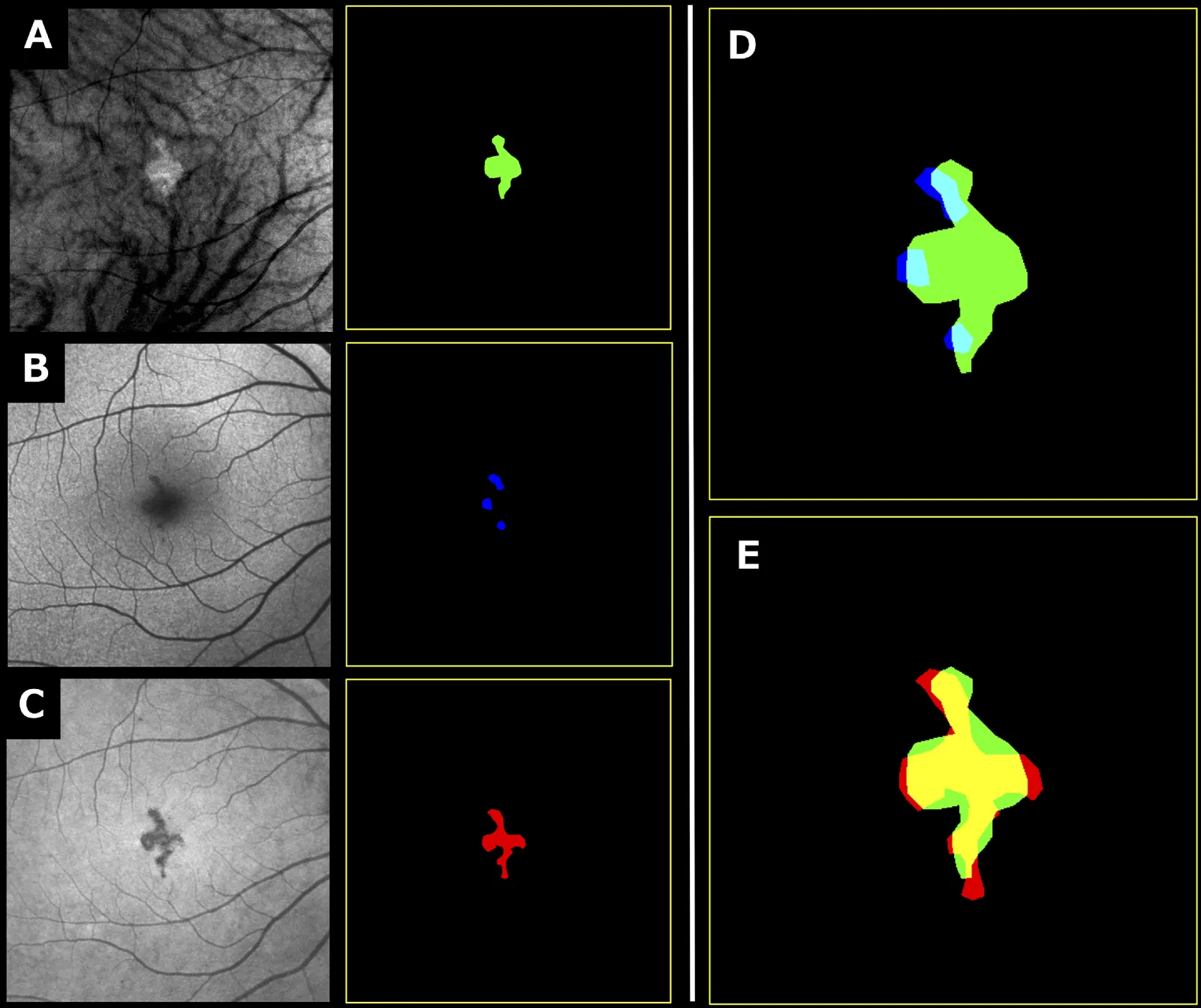
**Lesion segmentation and spatial overlap across imaging modalities.** (**A**) En face OCT image and the segmentation mask of the hyperreflective region corresponding to the FCE lesion (*green*; area, 0.368 mm^2^). (**B**) SWAF image and the corresponding segmentation mask of the abnormal autofluorescence region (*blue*; area, 0.057 mm^2^). (**C**) NIRAF image and the corresponding segmentation mask of the abnormal autofluorescence region (*red*; area, 0.310 mm^2^). (**D**) Overlay of the en face OCT mask (*green*) and the SWAF mask (*blue*). The overlapping region is shown in *cyan* (overlap area, 0.039 mm^2^; DSC, 0.183). (**E**) Overlay of the en face OCT mask (*green*) and the NIRAF mask (*red*). The overlapping region is shown in *yellow* (overlap area, 0.252 mm^2^; DSC, 0.744).

To assess the concordance between the FCE area measured on en face OCT and the corresponding regions of abnormal autofluorescence on each FAF modality, we calculated the Dice similarity coefficient (DSC). For each modality, the en face OCT-defined FCE area and the FAF abnormality were extracted as binary masks, overlaid, and their spatial overlap was quantified using the DSC, which ranges from 0 to 1, with higher values indicating greater spatial similarity (Fig. 3).^16^^,^^17^

The DSC was defined as follows:

$$\begin{array}{c} DSC\left(A,B\right)=\frac{2|A\cap B|}{|A|+|B|}, \end{array}$$

with A representing the en face OCT mask and B representing the FAF mask.

### Inter-Grader Image Comparison

All images were independently evaluated by two retina specialists (Y.N. and T.S.). When the graders disagreed on lesion delineation, a senior grader (S.K.) adjudicated the final determination. To assess reproducibility of each modality, intraclass correlation coefficients (ICCs) were calculated between the two graders, and Bland–Altman plots were generated^18^

### Statistical Analysis

Descriptive statistics for continuous variables were presented as mean ± standard deviation (SD). The Steel–Dwass test was used to compare FCE area measurements among the three imaging modalities (en face OCT, SWAF, and NIRAF). Differences in DSC between en face OCT and SWAF and between en face OCT and NIRAF were assessed using the Wilcoxon signed-rank test. All statistical analyses were performed using JMP Statistics version 17.0 Pro (JMP Statistical Discovery LLC, Cary, NC, USA), and a two-sided *P*-value < 0.05 was considered statistically significant.

## Results

### Patient Characteristics

A total of 20 eyes from 20 patients were included in the study. The mean age was 52.1 ± 16.7 years, and 10 patients (50%) were female. The mean best-corrected visual acuity at the initial visit was −0.036 ± 0.131 logMAR, corresponding to approximately 20/18 in Snellen equivalent. The mean axial length was 25.41 ± 1.36 mm. The mean subfoveal choroidal thickness was 220.7 ± 102.2 µm (Table 1).

**Table 1.** Baseline Characteristics

	Total (*n* = 20)
Age (years)	52.1 ± 16.7
Sex (male/female)	10/10
Baseline logMAR (Snellen)	−0.036 ± 0.131 (20/18)
Axial length (mm)	25.41 ± 1.36
Subfoveal choroidal thickness (µm)	220.7 ± 102.22

### Comparison of FCE Area Among Imaging Modalities

The mean FCE area measured on en face OCT was 0.708 ± 0.571 mm^2^, compared to 0.152 ± 0.203 mm^2^ on SWAF (*P* = 0.0003) and 1.174 ± 1.196 mm^2^ on NIRAF (*P* = 0.534) (Table 2). The mean DSC between en face OCT and SWAF was 0.160 ± 0.214, whereas that between en face OCT and NIRAF was significantly higher at 0.654 ± 0.187 (*P* < 0.001) (Table 3). Abnormal autofluorescence was not detected on SWAF in six eyes (30%), whereas NIRAF detected abnormal autofluorescence corresponding to the FCE area in all cases.

**Table 2.** Comparison of FCE Area Among Imaging Modalities

	En Face OCT	SWAF	NIRAF	*P* Value^a^	*P* Value^b^	*P* Value^c^
FCE area (mm^2^)	0.708 ± 0.571	0.152 ± 0.203	1.174 ± 1.196	0.0003	0.5337	<0.0001

FCE, focal choroidal excavation; NIRAF, near-infrared fundus autofluorescence; OCT, optical coherence tomography; SWAF, short-wavelength fundus autofluorescence.

a Comparison between En face OCT and SWAF by the Steel–Dwass test.

b Comparison between En face OCT and NIRAF by the Steel–Dwass test.

c Comparison between SWAF and NIRAF by the Steel–Dwass test.

**Table 3.** Comparison of Dice Coefficients Between En Face OCT and Autofluorescence Modalities

	En Face OCT Vs. SWAF	En Face OCT Vs. NIRAF	*P*-Value
Dice coefficient	0.160 ± 0.214	0.654 ± 0.187	<0.0001

### Intergrader Agreement for FCE Area Measurements

**Figure 4.**
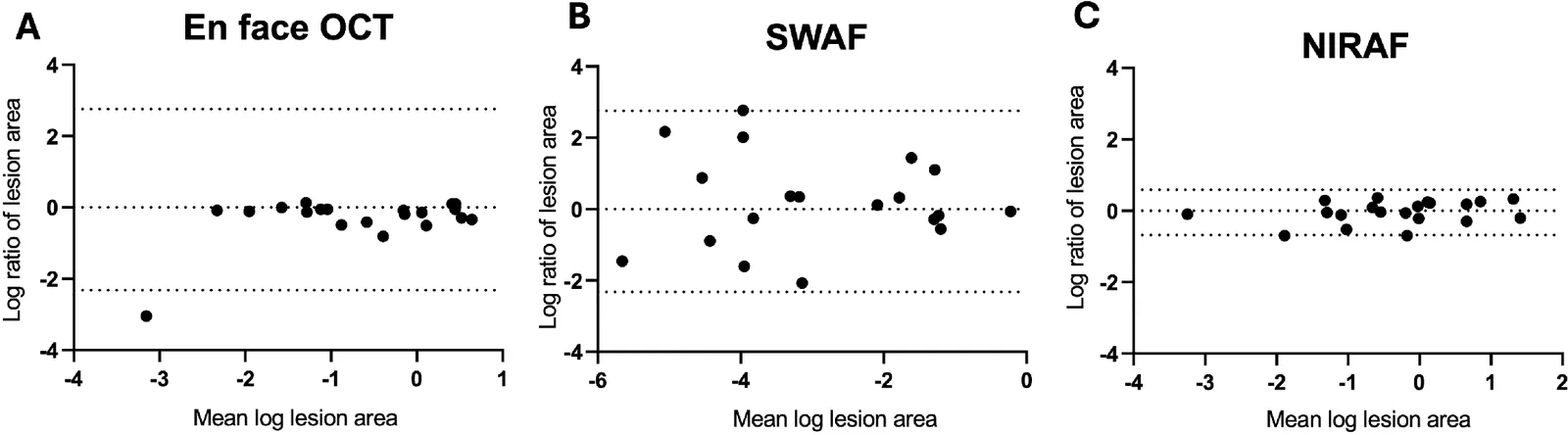
**Bland–Altman plots for intergrader agreement.** Bland–Altman plots show the difference in log-transformed lesion area measurements between two graders (y-axis; Grader A − Grader B) against their mean (x-axis). The central horizontal line indicates the mean difference, and the upper and lower horizontal lines indicate the 95% limits of agreement. Intergrader differences were assessed on the log scale. The spread of intergrader differences was smallest for NIRAF (**C**), intermediate for en face OCT (**A**), and largest for SWAF (**B**) (mean ± SD: −0.045 ± 0.322, −0.323 ± 0.683, and +0.219 ± 1.295, respectively). One SWAF case, one retinal specialist judged that no abnormal autofluorescence was present. Because a log-transformed value could not be obtained for this eye, it was excluded from the Bland–Altman analysis.

Intergrader agreement for FCE area measurements was evaluated using the ICC with a two-way random-effects model (ICC [2,1]). The ICC was 0.899 (95% confidence interval [CI], 0.765–0.959) for en face OCT, 0.838 (95% CI, 0.636–0.933) for SWAF, and 0.931 (95% CI, 0.835–0.972) for NIRAF. Bland–Altman plots were generated to visualize intergrader agreement for en face OCT and each autofluorescence modality. To account for baseline differences across modalities, measurements were log-transformed before analysis. The mean difference (± SD) between graders was −0.32251 ± 0.68270 for en face OCT, 0.21930 ± 1.29547 for SWAF, and −0.04479 ± 0.32227 for NIRAF, indicating the smallest variability for NIRAF (Fig. 4). The single en face OCT case showing a large intergrader discrepancy was an eye in which the FCE area on en face OCT was very small, making delineation difficult. In SWAF, multiple eyes showed more than a twofold difference between graders; these were eyes in which the excavation region largely overlapped the physiologically hypoautofluorescent foveal area, complicating boundary definition. In addition, in one SWAF case, one retinal specialist judged that no abnormal autofluorescence was present. Because a log-transformed value could not be obtained for this eye, it was excluded from the Bland–Altman analysis.

## Discussion

Using the FCE area defined on en face OCT as the anatomical reference, we performed a registration-based quantitative comparison of the spatial concordance of autofluorescence abnormalities on NIRAF and SWAF with the structural excavation. NIRAF demonstrated significantly greater spatial concordance with the OCT-defined FCE area than SWAF (*P* < 0.0001). In addition, NIRAF detected corresponding autofluorescence abnormalities in all eyes, whereas no abnormality was identified on SWAF in 30% of eyes. Furthermore, the area of abnormal autofluorescence differed between modalities, being larger on NIRAF (1.174 ± 1.196 mm^2^) than on SWAF (0.152 ± 0.203 mm^2^). Intergrader agreement was excellent across modalities (ICC [2,1] for en face OCT, SWAF, and NIRAF: 0.899, 0.838, and 0.931, respectively), supporting the robustness and clinical applicability of these measurements. The present study should be interpreted as a quantitative extension of prior qualitative and case-based observations rather than as the first description of NIRAF abnormalities in FCE.^12^^,^^13^ Although previous reports suggested that NIRAF may depict FCE lesions more clearly than SWAF, the present study provides registration-based quantitative evidence that NIRAF abnormalities show greater spatial concordance with the en face OCT-defined FCE area and highly reproducible measurements compared with SWAF abnormalities.

Ocular melanin absorbs light and functions as a natural antioxidant, thereby protecting ocular tissues—particularly the RPE and choroid—from phototoxic and oxidative damage. A reduction in choroidal melanosomes may render the choroid more susceptible to photo-oxidative injury, which in turn can contribute to the well-recognized vicious cycle of choroidal vascular degeneration in aging and disease.^19^^,^^20^ NIRAF is a noninvasive modality that enables in vivo visualization of predominantly melanin-related autofluorescence within the RPE and choroid using near-infrared excitation, in contrast to SWAF, which predominantly reflects lipofuscin.^21^^,^^22^ NIRAF is clinically used as a melanin-sensitive imaging modality in a variety of conditions.^21^ For example, pigmented choroidal nevi appear hyperautofluorescent on NIRAF owing to increased melanin, whereas in disorders with loss or thinning of melanin, such as choroideremia or oculocutaneous albinism, the NIRAF signal is markedly reduced or even absent.^10^^,^^11^^,^^23^ FCE is a disorder characterized by a localized concavity of the choroid, first described by Jampol et al. in 2006.^1^ Although the pathogenesis and clinical significance of FCE remain incompletely understood, our finding that NIRAF shows hypoautofluorescence corresponding to the excavation suggests that this modality captures a reduction in melanocytes or melanin content in regions of choroidal loss within FCE. Together with the quantitative results described above, these observations indicate that NIRAF is particularly well suited for depicting structural alterations associated with FCE.

**Figure 5.**
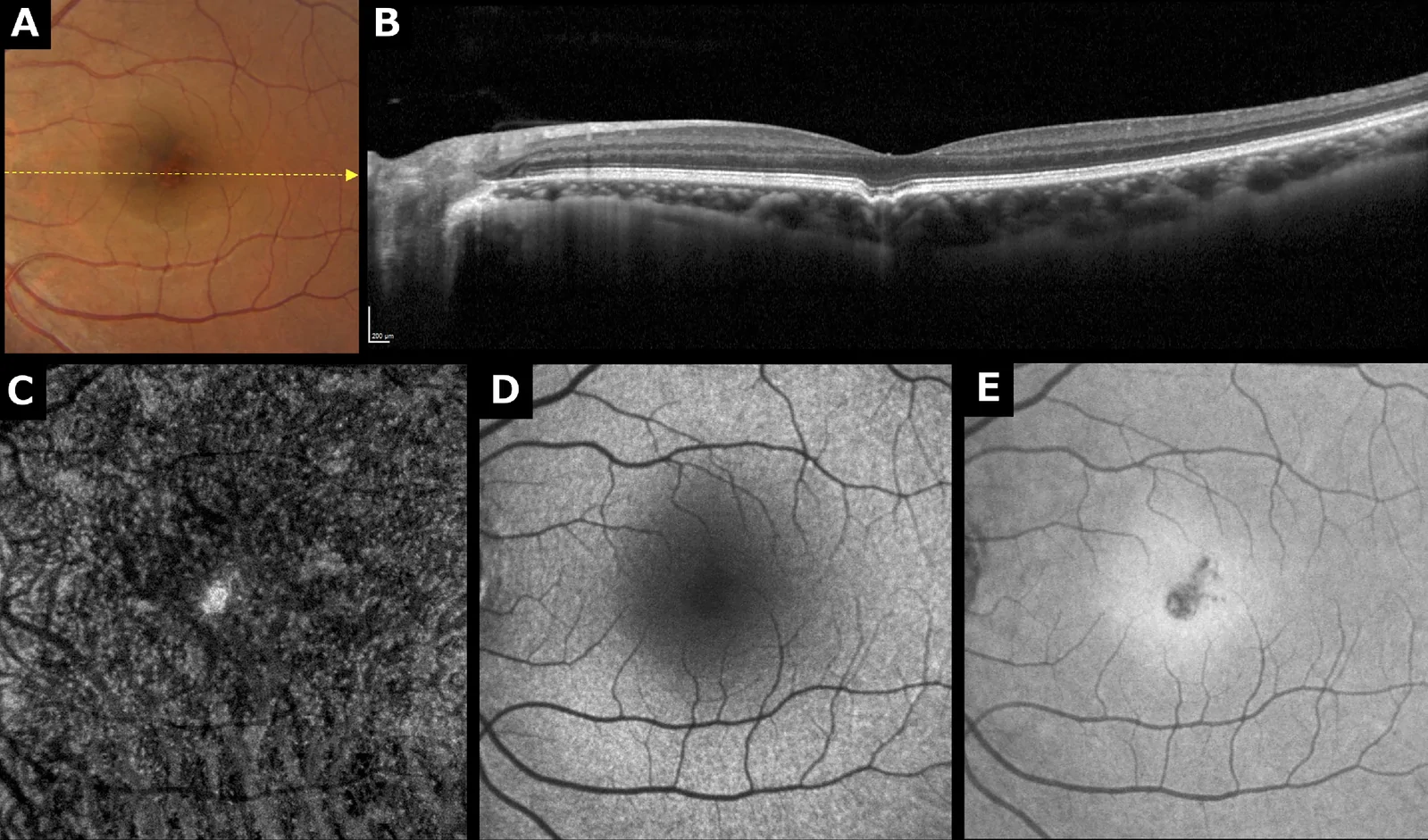
**Representative multimodal images of FCE.** (**A**) Color fundus photograph. The *yellow line* indicates the location of the OCT B-scan. (**B**) OCT B-scan demonstrating FCE. (**C**) En face OCT image showing the FCE lesion as a hyperreflective region. (**D**) SWAF image in which the abnormal autofluorescence area appears limited and partially delineated compared with the FCE lesion shown in (**C**). (**E**) NIRAF image in which the abnormal autofluorescence area appears more extensive than on SWAF and more closely corresponds to the FCE lesion shown in (**C**).

In this study, NIRAF demonstrated significantly greater spatial concordance with en face OCT than SWAF and all FCE lesions were identified on NIRAF (Fig. 5). Several factors may account for the inferior performance of SWAF. In the conforming-type FCE that we examined, structural damage to the photoreceptors and RPE is generally limited, so the lesions may not generate sufficient lipofuscin-related signal to produce a conspicuous SWAF abnormality. In addition, FCE frequently arises in the foveal or parafoveal region, where dense macular pigment strongly attenuates blue-light excitation, further hampering the evaluation of SWAF images. By contrast, NIRAF is less affected by macular pigment and directly reflects melanin-related alterations, which likely contributes to its superior lesion detectability. Importantly, the absence of a detectable SWAF abnormality should not be interpreted as evidence of preserved RPE metabolic function or normal lipofuscin turnover. Because SWAF signals are influenced by macular pigment attenuation, subtle lipofuscin-related abnormalities may have remained below the detection threshold. Green-light autofluorescence (GAF), which is less affected by macular pigment absorption, might have provided additional information in this regard; however, it was not routinely acquired in this retrospective study.^9^

In some eyes, the NIRAF abnormality extended beyond the boundary of the excavation defined on en face OCT. One possible explanation is that hemodynamic and tissue alterations in the perifocal choroid may become detectable before structural remodeling is sufficient to produce a morphologically apparent change in the choroidal contour on OCT. Previous studies have reported impaired choroidal circulation and reduced retinal functional responses in eyes with FCE.^24^ In addition, OCT angiography has demonstrated abnormal choriocapillaris flow signals surrounding the flow signal–absent excavation, suggesting that choroidal hemodynamic alterations may extend beyond the structurally defined FCE area.^25^ Such perifocal hemodynamic abnormalities may be accompanied by alterations in melanocyte density, melanin content, or melanin-related tissue organization within the RPE and choroid, thereby producing an abnormal NIRAF signal despite the absence of an overt excavation on OCT. A similar dissociation between structurally defined and biologically affected boundaries has been described in geographic atrophy secondary to AMD, in which retinal dysfunction may extend beyond the clinically apparent atrophic margin and lesion enlargement subsequently proceeds from the junctional zone.^26^^,^^27^ By analogy, the NIRAF abnormality surrounding FCE may represent a perifocal region affected by the underlying disease process before the development of a clearly detectable structural excavation. However, because the present study was cross-sectional, it remains unclear whether these perifocal NIRAF abnormalities precede subsequent enlargement of the excavation or instead represent secondary changes surrounding an established lesion. Moreover, MNV has been reported to arise at the margin or along the slope of FCE.^28^^,^^29^ Whether NIRAF abnormalities outside the OCT-defined excavation identify regions at increased risk of future structural progression or MNV development should therefore be investigated in longitudinal studies.

In this study, two retinal specialists independently graded the extent of autofluorescence abnormalities on SWAF and NIRAF, as well as the anatomical reference images obtained from en face OCT. Intergrader measurement error was small for all three modalities, indicating that each provides highly reproducible and clinically applicable measurements. The intergrader agreement, as assessed by ICC[2,1], was highest for NIRAF, and Bland–Altman plots also demonstrated the smallest intergrader differences for NIRAF. Taken together with its high sensitivity for detecting FCE lesions, relative insensitivity to macular pigment, and ease of measurement, these findings support NIRAF as the preferred modality for quantitative assessment of FCE-related changes.

This study has several limitations. First, the sample size was relatively small (20 eyes), and further data accumulation is warranted. Nevertheless, this represents one of the largest quantitative comparisons to date, demonstrating a statistically significant advantage of NIRAF over SWAF for depicting conforming-type FCE. Second, GAF was not routinely acquired and could not be evaluated because of the retrospective study design. Because GAF is less affected by macular pigment absorption than SWAF, the absence of GAF limits our ability to determine whether the greater spatial concordance of NIRAF reflects its melanin sensitivity, reduced macular pigment interference, or both. Future prospective studies incorporating GAF are warranted. Third, this was a cross-sectional study; therefore the longitudinal implications of NIRAF abnormalities remain unknown. Prospective follow-up studies are needed to determine whether NIRAF-detected alterations predict subsequent MNV development or lesion enlargement.

## Conclusions

NIRAF abnormalities showed greater spatial concordance with the en face OCT-defined FCE area and were more consistently detectable than SWAF abnormalities, while measurements on NIRAF also demonstrated high intergrader reproducibility. These findings support the use of NIRAF for characterizing and quantitatively assessing FCE-associated autofluorescence changes. NIRAF may also facilitate longitudinal monitoring of perifocal autofluorescence changes; however, prospective studies are needed to clarify whether such changes relate to lesion evolution or subsequent MNV development.
